# Mitonuclear coevolution as the genesis of speciation and the mitochondrial DNA barcode gap

**DOI:** 10.1002/ece3.2338

**Published:** 2016-07-22

**Authors:** Geoffrey E. Hill

**Affiliations:** ^1^ Department Biological ScienceAuburn University331 Funchess HallAuburnAlabama36849‐5414

**Keywords:** Electron transport chain, mitonuclear coadaptation, neutral theory, oxidative phosphorylation, parapatric speciation

## Abstract

Mitochondrial genes are widely used in taxonomy and systematics because high mutation rates lead to rapid sequence divergence and because such changes have long been assumed to be neutral with respect to function. In particular, the nucleotide sequence of the mitochondrial gene cytochrome *c* oxidase subunit 1 has been established as a highly effective DNA barcode for diagnosing the species boundaries of animals. Rarely considered in discussions of mitochondrial evolution in the context of systematics, speciation, or DNA barcodes, however, is the genomic architecture of the eukaryotes: Mitochondrial and nuclear genes must function in tight coordination to produce the complexes of the electron transport chain and enable cellular respiration. Coadaptation of these interacting gene products is essential for organism function. I extend the hypothesis that mitonuclear interactions are integral to the process of speciation. To maintain mitonuclear coadaptation, nuclear genes, which code for proteins in mitochondria that cofunction with the products of mitochondrial genes, must coevolve with rapidly changing mitochondrial genes. Mitonuclear coevolution in isolated populations leads to speciation because population‐specific mitonuclear coadaptations create between‐population mitonuclear incompatibilities and hence barriers to gene flow between populations. In addition, selection for adaptive divergence of products of mitochondrial genes, particularly in response to climate or altitude, can lead to rapid fixation of novel mitochondrial genotypes between populations and consequently to disruption in gene flow between populations as the initiating step in animal speciation. By this model, the defining characteristic of a metazoan species is a coadapted mitonuclear genotype that is incompatible with the coadapted mitochondrial and nuclear genotype of any other population.

## Introduction

The concept of species is fundamental in biology. Despite a century of discussion and debate, however, the nature of species and the process by which speciation occurs remain contentious (Price 2007; Butlin et al. 2012; Nosil 2012). With no agreement on the process of speciation but with a pressing need to inventory biodiversity, molecular biologists have sought a means to identity species using DNA sequences – the so‐called DNA barcoding (Hebert et al. 2003a; Kress et al. 2014). DNA barcoding is generally presented as a means to automate the recognition of previously defined taxa, and a 648‐base pair region of the cytochrome *c* oxidase subunit one (COX1) gene has proven highly effective in correctly distinguishing among metazoan species (Hebert et al. 2003b; Dasmahapatra and Mallet 2006; Bucklin et al. 2011). The sorting of animal species by COX1 genotype is enabled by a barcode gap whereby individuals in a species are more similar to each other in COX1 sequence than they are to individuals in any other species (Hebert et al. 2003a; Bucklin et al. 2011). In the great majority of cases, the metazoan populations recognized as distinct by COX1 sequence groupings are congruent with already recognized species (Tavares et al. 2011) or barcode gaps lead to the recognition of new species upon further study (Hebert et al. 2004; Bickford et al. 2007).

The view of COX1 that is commonly articulated by taxonomists is that it is a molecular marker chosen to play the role of DNA barcode because it is a gene found in all animals that can be amplified by a few primer sets (Yu et al. 2012; Deagle et al. 2014). The implication of this view is that there is nothing particularly special about COX1 with regard to speciation – It is a gene that accumulates random mutations following isolation of gene pools like many other, particularly mitochondrial, genes (Seberg et al. 2003; Tautz et al. 2003; Moritz and Cicero 2004; Hickerson et al. 2006). Within the context of DNA barcodes, there is some discussion that the reason that COX1 is so conserved is that it plays a vital role in cellular respiration and hence is subject to strong stabilizing selection (Stoeckle and Thaler 2014) and that it is coadapted with the nuclear genes with which it interacts (Hebert et al. 2003b). Nevertheless, there has been little consideration of the enormous implications for theories of animal speciation of such an effective mitochondrial barcode gene (Lane 2009).

In this essay, I extend the argument that COX1 is so successful as a genetic marker of the boundaries between closely related animal species because mitochondrial genes are directly involved in the process of speciation (Gershoni et al. 2009; Lane 2009). I begin by establishing that COX1 genotypes are very effective at delimiting species boundaries within taxa of complex animals. I then provide an overview of the architecture of the core mechanism for cellular respiration in mitochondria that necessitates perpetual coevolution between mitochondrial genes (mt genes) and nuclear genes that express in the mitochondrial (N‐mt genes). I emphasize the hypothesis that coevolution between mt and N‐mt genes rapidly generates barriers to gene flow as the initiating step in speciation. Such divergence in coadapted mt and N‐mt genotypes can emerge in allopatry through genetic divergence that is neutral with respect to changes in environmental adaptation. Alternatively, divergence of coadapted mt and N‐mt genotypes can theoretically emerge in parapatry via adaptive divergence (i.e., ecological speciation) when small changes to the electron transport chain (ETC) – or to the mechanisms that produce ETC subunits – underlie adaptation to a discrete set of novel environmental conditions. By this mitonuclear compatibility hypothesis for speciation, the initiating step in speciation is the evolution of mitonuclear incompatibilities that create barriers to gene flow (Burton and Barreto 2012), leaving mitochondrial genotype as an unambiguous marker of species identity.

### How effective is the COX1 barcode gap in delimiting species boundaries?

There is an ongoing debate regarding the value of the COX1 gene sequence as a DNA barcode to delimit the boundaries of species, with some authors championing the approach (Kerr et al. 2007; Bucklin et al. 2011) and others arguing that reliance on a single gene to delimit species will frequently fail to identify closely related taxa (Will et al. 2005; Hickerson et al. 2006). Some of the reservations by taxonomists about the application of DNA barcoding arise from concern that automating taxonomy will remove the need for taxonomists and undercut support and funding for museum scientists (Will et al. 2005). However, debate regarding the efficacy of COX1 barcoding also arises from empirical assessments of COX1 sequence variation relative to species boundaries in various groups of animals (Meier et al. 2006; Elias et al. 2007; Whitworth et al. 2007).

In a recent review of the efficacy of COX1 as a barcode as applied to all major taxonomic groups of marine animals, Bucklin et al. (2011) concluded it worked well for all marine animals with the exception of the “problem children”: corals, sea anemones, and sponges. Interestingly, COX1 barcoding seems to fail for multicellular animals lacking complex nervous systems or sustained movement; the need for neurogenesis and real‐time production of ATP for movement may increase selection on mitonuclear coadaptation and hence lead to clearer divergence of coadapted set of mt and N‐mt genes between species. Perhaps for the same reason, COX1 is not effective as a universal barcode gene for plants or fungi (Kress et al. 2005; Eberhardt 2012).

The primary difficulty in judging of efficacy of the COX1 barcode gene among animals is the variable quality of current taxonomies. In many cases, taxonomies were constructed over the past century based on morphology, sometimes with minimal sampling (Dubois 2007; Morrison et al. 2009). Thus, it is rarely possible to distinguish failures of DNA barcoding due to mitochondrial introgression or incomplete lineage sorting from failures that are a consequence of inaccurately drawn species boundaries. (See Mutanen et al. ([Link]) for an attempt to disentangle factors contributing to apparent mismatches of COX1 barcode gaps and species boundaries in Lepidoptera.) For instance, COX1 genotyping revealed 10 distinct taxa within what had long been considered a single species of a common and well‐known skipper butterfly, *Astraptes fulgerator* (Hebert et al. (2004) but see also Brower (2006)). An even greater diversity of cryptic species of parasitic flies was discovered using COX1 barcoding within what was thought to be one taxon (Scheffers et al. 2012). Without detailed studies of host‐plant and host‐caterpillar differentiation within these cryptic taxa to support the species boundaries revealed by DNA barcoding, the efficacy of COX1 barcoding in these groups would have been considered dismal. Many animal taxa likely harbor cryptic species (Bickford et al. 2007), leaving assessments of the efficacy of COX1 barcoding uncertain in many taxa.

Birds are the best taxon to assess the efficacy of DNA barcoding because they are the most comprehensively described animal taxon (Scheffers et al. 2012). Class Aves has long been the model taxon for the study of speciation in the field because birds are easy to observe, and they use primarily human‐perceptible visual and acoustical displays in sexual signaling (Price 2007). Over large areas of the planet, the distributions of bird populations and the frequency with which avian taxa form hybrid pairs and produce hybrid young is well documented (Price 2007). Disagreements about avian taxonomy tend to be based on differences in species concepts rather than lack of information about phylogeography (Remsen et al. 2010). In assessments of hundreds of species of birds from North America, South America, and the Palearctic, COX1 barcoding clustered individuals into groups that conformed to conventional avian species in more than 94% of the cases (Aliabadian et al. 2009; Baker et al. 2009; Kerr et al. 2009a, 2009b *;* Kerr 2011; Tavares et al. 2011; Stoeckle and Thaler 2014). Most of the “failures” of DNA barcoding in these avian studies involved taxa in which species boundaries have been long debated, such as large gulls, or were due to divergences within taxa recognized as single species that suggested the presence of cryptic species. The true test of the efficacy of COX1 barcoding, however, hinges on its ability to separate closely related sister taxa. In a study of the 60 sister pairs of bird species for which there was sufficient COX1 sequence data available, Tavares and Baker (2008) found that all 60 previously recognized species boundaries were diagnosable by COX1 DNA barcode. Thus, for class Aves, which is the best‐known animal taxon, the COX1 DNA barcode is very effective at delimiting species boundaries. The question is, how does a DNA barcode gap between species arise?

### Mitonuclear coadaptation

Coadaptation of the products of the mitochondrial genome and nuclear genome is fundamental to the fitness of all eukaryotes (Lane 2011; Bar Yaacov et al. 2012). The proteome of the mitochondria of animals is composed of more than 1500 products of the nuclear genome (Calvo and Mootha 2010). These N‐mt genes are joined in the mitochondria by the products of the 37 mt genes that code for proteins in the ETC or for transcriptional or RNA components of translational machinery needed to produce respiratory chain proteins (Levin et al. 2014; Hill 2015). N‐mt and mt genes function in close and highly coadapted association in forming the complexes of the respiratory chain that carry out oxidative phosphorylation (OXPHOS) and that produce most of the energy for eukaryotic life (Rand et al. 2004; Bar‐Yaacov et al. 2012). The need for mitonuclear compatibility is fundamental (Mishmar et al. 2006; Hill 2015). Any incompatibility of mt and N‐mt genes leads to inefficiency in electron flow down the respiratory chain, which causes catalytic sites to become more reduced and results in the release of free radicals and a reduction in ATP production (Lane 2011, 2015).

The coadaptation of sets of mt and N‐mt genes is highly specific to species. In cell cultures in which nuclear DNA from one species is forced to cofunction with mt DNA from a closely related species in cybrid cells, the efficiency of OXPHOS is invariably significantly compromised (Kenyon and Moraes 1997; Barrientos et al. 2000; McKenzie et al. 2003). In marine copepods *Tigriopus californicus*, crossing animals from geographically isolated populations resulted in a 40% loss of ATP production in F2 population hybrids (Ellison and Burton 2006; Burton et al. 2013), with fitness loss linked specifically to loss of function in complexes I, III, and IV of the ETC due to incompatibilities between maternal mt genes and paternal N‐mt genes (Ellison and Burton 2008). Similar mitonuclear incompatibilities with loss of fitness have been observed in carefully manipulated hybrid crosses in *Drosophila* (Sackton et al. 2003; Meiklejohn et al. 2013), parasitoid wasps (Ellison et al. 2008), and fish (Bolnick et al. 2008) in which mt genes from one species are expressed with N‐mt genes from another, closely related species. Many different mitochondrial and nuclear genes can be involved in hybrid incompatibilities, including subunits of the ETC and components of transcriptional and translational mechanisms (Burton and Barreto 2012; Meiklejohn et al. 2013; Levin et al. 2014; Hill 2015), but cytochrome *c* oxidase (complex IV) and cytochrome *c* are frequently implicated in incompatibilities (Rawson and Burton 2002; Sackton et al. 2003; Harrison and Burton 2006). The key point for exploring the connections between a mitochondrial DNA barcode and the process of speciation is that mitochondrial genes often play a central role in hybrid incompatibilities (Gershoni et al. 2009; Chou and Leu 2010; Burton and Barreto 2012; Burton et al. 2013).

### Mitonuclear coadaptation and speciation

Conventional models of speciation cannot fully account for the barcode gap between closely related species that makes COX1 such a successful barcode gene (Hickerson et al. 2006; Rubinoff et al. 2006). By classical Bateson–Dobzhansky–Muller models of allopatric speciation, cladogenesis begins when two or more populations are separated in space or time such that gene flow among the populations is disrupted (Coyne and Orr 2004; Price 2007). In isolation, each gene pool undergoes independent changes to its gene sequence via both selection and drift. Eventually the populations diverge to a point where they are diagnosable as new taxa. Under some species concepts, speciation is achieved with diagnosability; in others, speciation requires reproductive isolation after secondary contact (Coyne and Orr 2004; Price 2007).

From a neutral process of lineage sorting, there should emerge a more‐or‐less constant accumulation of synonymous nucleotide changes at a rate dependent on the mutation rate and population size for the species (Crow and Kimura 2009). For COX1 barcode gaps to accurately predict species boundaries, COX1 genotype must completely sort among lineages; in other words, fixed differences must evolve between species in the COX1 nucleotide sequences such that these differences are shared among individuals within the species but not with any individuals outside the species boundary (Fujisawa and Barraclough 2013). According to neutral models, the time required for complete lineage sorting is equal or >2.6 times the effective population size (Nei 1987; Hudson and Turelli 2003). From a basic neutral theory model of speciation, simulations using various population sizes estimated that it would take approximately 4 million generations (hence a minimum 4 million years for birds or other large metazoans) for populations to diverge sufficiently in COX1 for accurate sorting of species (Hickerson et al. 2006). This estimate assumes a 10× rule – 10 times the amount of variation between species versus within species is required before species discrimination is achieved. A less stringent cutoff for COX1 divergences (e.g., a 2× rule) reduces the time needed for speciation, but it is still on the order of millions of years. However, predictions of neutral models for genetic divergence of COX1 genes are not supported in birds. Regardless of population sizes (Stoeckle and Thaler 2014) or estimated time since isolation (Hebert et al. 2003b; Baker et al. 2009; Kerr et al. 2009b), divergences in the COX1 gene in birds are congruent with taxonomist‐determined species boundaries.

Under the conventional Bateson–Dobzhansky–Muller models of speciation, with divergence of genotype via neutral processes, there is no mechanism for a particular gene in either the nuclear or the mitochondrial genome to consistently diverge in concert with speciation events. For the COX1 DNA barcode to work under neutral theory, there must be sufficient time for lineages to sort via random accumulation of neutral mutations (Hudson and Coyne 2002) and gene flow among species must be negligible (Lohse 2009). In birds, however, huge radiations of species with unique COX1 nucleotide sequences evolved within a time period significantly less than the time required for complete lineage sorting under neutral theory. For instance, in a study of radiations of South American birds, it was estimated that 75% of species diverged ≤2.6 million years ago (Smith et al. 2014). Avian speciation is even more rapid at higher latitudes where all sister taxa were estimated to have diverged <1 million years ago (Weir and Schluter 2007). For these numerous, recently diverged species, any single gene is predicted to be a poor marker of species boundaries (Hickerson et al. 2006). Indeed, for many avian taxa, species cannot be delimited by any single nuclear gene (Toews and Brelsford 2012), yet, paradoxically, COX1 works remarkably well as a DNA barcode for birds, consistently distinguishing between closely related, sister taxa of birds (Tavares and Baker 2008). An explanation for why the sequence of a specific gene coincides so consistently with species boundaries is that the barcode gene, or the other mt genes to which it is linked, plays a role in the process of speciation (Gershoni et al. 2009; Lane 2009).

## Speciation Via Mitonuclear Coevolution to Maintain Coadaptation

Rapid divergence of coadapted mt and N‐mt genes between isolated populations via mitonuclear coevolution was proposed and outlined in detail in Gershoni et al. (2009), Chou and Leu (2010), and Burton and Barreto (2012). The simplest form of such population divergence is driven by selection to maintain OXPHOS function through mitonuclear coadaptation independent of adaptive changes in cellular respiration. Divergent mitonuclear coevolution is inevitable whenever populations are isolated because the nonrecombining and haploid genomes of mitochondria are subject to high mutation rates in metazoans (Lynch 2010). As a consequence, slightly deleterious mutations perpetually accumulate in the mitochondrial genome (Lynch and Blanchard 1998; Neiman and Taylor 2009), and because all mitochondrial genes code for OXPHOS function, an accumulation of deleterious mutations in the mitochondrial genome erodes OXPHOS function resulting in loss of fitness (Wallace 2010). Growing evidence suggests that N‐mt genes can evolve so as to compensate for the deleterious effects of mutations in mt genes, thereby reversing deterioration of OXPHOS (Mishmar et al. 2006; Barreto and Burton 2013; Havird et al. 2015; van der Sluis et al. 2015). mt genes can also potentially compensate for changes in deleterious changes in N‐mt genes. Such Red Queen coevolutionary interactions between codependent mt and N‐mt genes lead to perpetual and unpredictable divergence and consequent incompatibilities between populations in mitonuclear gene complexes (Burton and Barreto 2012; Hill 2014; Levin et al. 2014; Chou and Leu 2015). Divergence in coadapted mitonuclear complexes between populations would, in turn, produce mitonuclear incompatibilities in hybrid offspring creating a postzygotic isolating mechanism (Gershoni et al. 2009; Chou and Leu 2010; Burton and Barreto 2012).

Under this model, speciation is the process of divergence of populations specifically in coadapted mitonuclear genotypes. It follows that a species can be defined as a population that is reproductively isolated from other populations by incompatibilities in uniquely coadapted mt and N‐mt genes. This mitonuclear compatibility species concept implicates mt genes – along with that subset of N‐mt genes with which mt genes cofunction – as central players in the process of speciation and the defining characteristics of species. This process of rapid, non‐neutral divergence in mitochondrial genotype in isolated populations provides an explanation for why mitochondrial genotype diagnoses species. This model for speciation could be thought of as a refinement of classical biological speciation (Mayr 1942) reinforced by Dobzhansky–Muller incompatibilities (Dobzhansky 1936; Muller 1942). Burton and Barreto (2012) provide a detailed overview of why coadapted mitochondrial and nuclear genes are likely to play a disproportionate role in the generation of Dobzhansky–Muller incompatibilities in genetically diverging populations.

### Complications from inherited symbionts

Inherited symbionts and particularly *Wolboachia* bacteria may enable introgression of diverged mt genotypes in arthropods and therefore eliminate a mt DNA barcorde gap between species (Hurst and Jiggins 2005). The scenario for such symbiont‐enabled mt introgression is a hybridization event that transfers both a novel symbiont and a novel mt haplotype into a population. Even if the mt genotype has reduced fitness because of poor coadaptation with N‐mt genes of the new population, it can theoretically spread in the new population if there is strong selection for spread of the symbiont; because of maternal cotransmission of both the mt genotype and the symbiont, the mt genotype can hitchhike to fixation if the symbiont spreads to infect all individuals in the new population (Charlat et al. 2009; Klopfstein et al. 2016). The result can be loss of a DNA barcode gap between populations that might otherwise be quite divergent, and the evolution of a population with poorly coadapted mt and N‐mt genes. Mitonuclear incompatibility should create strong selection for coevolution to restore coadaptation (Hill 2014) or for the introgression of N‐mt genes that are coadapted with mt haplotype (Beck et al. 2015; Boratyński et al. 2015). There are at present few well‐documented examples of mitochondrial introgression driven by Wolbachia infection (Klopfstein et al. 2016) and the success of DNA barcoding in delimiting species boundaries in many arthropod taxa suggests that such introgression may not be very common in arthropods (Smith et al. 2012).

### COX as a key OXPHOS regulator

In the model described, mitonuclear coevolution is driven by selection to maintain current function in cellular respiration; no change in environmental adaptation is invoked. I propose, however, that speciation can also occur through adaptive divergence of coadapted mitonuclear genes and that COX genes are likely to be common targets of such adaptive evolution because cytochrome *c* oxidase occupies a key rate‐controlling position in the ETC (Villani and Attardi 1997; Piccoli et al. 2006; Pacelli et al. 2011; Arnold 2012). Complex IV is the terminus of the respiratory chain of mitochondria, catalyzing the transfer of electrons from reduced cytochrome *c* to oxygen and in turn reducing oxygen to water. Energy from this redox reaction is used to pump protons across the inner mitochondrial membrane. In this way, cytochrome *c* oxidase plays a central role in creating the inner membrane potential that is necessary for aerobic respiration (Pierron et al. 2012). Only the mitochondrial‐encoded COX proteins – subunits 1, 2, and 3 – are essential for the catalytic function of cytochrome *c* oxidase (Pierron et al. 2012; Ramzan et al. 2012). Prokaryotes have only these three subunits, and these are the subunits that actually transfer electrons, pump protons, and reduce oxygen to water (Pierron et al. 2012). The many additional subunits that eukaryotes have added to cytochrome *c* oxidase (e.g., 10 in mammals) are nuclear encoded, and apparently evolved to provide critical regulatory control of the three catalytic subunits, establishing the balance between ROS, heat, and energy production in variable environments (Pierron et al. 2012; Ramzan et al. 2012). Thus, the N‐mt genes of cytochrome *c* oxidase have intimate physical and functional connection to the mt‐encoded catalytic subunits and play a vital role in better adapting cytochrome *c* oxidase to the specific environmental conditions of the organism (Pierron et al. 2012; Ramzan et al. 2012; Hill 2015). Interestingly, COX1 and other mitochondrial‐encoded cytochrome *c* oxidase genes are the least variable of all mitochondrial genes (Pesole et al. 1999; da Fonseca et al. 2008), apparently subject to strong purifying selection and functional constraint (Kerr 2011).

Cytochrome *c* oxidase is unique among respiratory chain complexes in having multiple isoforms of the nuclear‐encoded components (Hüttemann et al. 2001). Different isoforms of subunits occur in different tissues (Kadenbach et al. 2000) and at different developmental stages (Bonne et al. 1993). Isoforms enable control of ATP production and the membrane potential across variable conditions in different organs and tissues within an individual (Pierron et al. 2012; Ramzan et al. 2012). The existence of subunit isoforms exclusively for cytochrome *c* oxidase among the five OXHPOS complexes underscores the importance of local adaptation of complex IV activity among both external and internal environments for efficient energy production and control of ROS leakage (Pierron et al. 2012). I propose that it is no coincidence that the rate‐controlling gene in the ETC that can enable respiratory adaptation to novel environments is also an effective DNA barcode for animals.

### Speciation via adaptive divergence in mitonuclear respiratory genes

**Figure 1:**
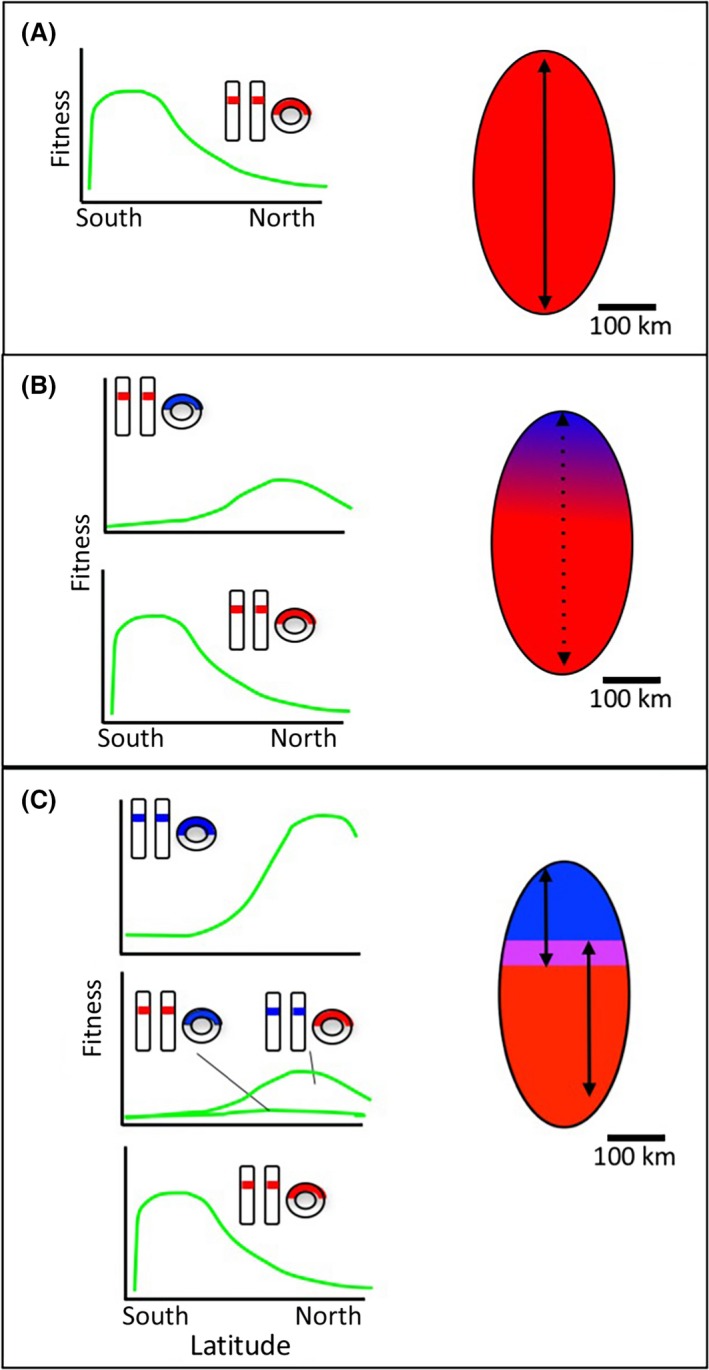
Speciation via adaptive divergence in genotypes of mt and N‐mt genes that interact with mt. Graphs show hypothetical fitness curves across a latitudinal gradient for different mt/N‐mt genotypes. The ovals at right represent hypothetical ranges of a parent and then daughter species with the map oriented with north to the top. N‐mt genotype is represented by colored bars within chromosomes while mt genotype is represented by colored section of the circular mt genome. (A) The ancestral mt genotype (red) has high fitness in the south and low fitness in the north. The ancestral N‐mt gene (red) enables the function of the ancestral mt. (B) A mutation produces a novel mt genotype (blue) that has high fitness in the north and low fitness in the south. Its function is supported by the ancestral N‐mt gene (red), but it fails to achieve fitness in the north as high as the ancestral mt genotype in the south. Natural selection creates a cline in mt genotypes, but gene flow erodes local adaptation. (C) A mutation leads to a novel N‐mt genotype (blue) that better supports the function of the cold adapted mt gene (blue) and raises the fitness of individuals in cold climate. The new N‐mt gene has low compatibility with the ancestral mt genotype (red), creating a barrier to gene flow via hybrid dysfunction and initiating speciation.

Adaptive divergence in mitochondrial genotype provides a robust explanation for COX1 divergence in sister taxa separated by relatively few generations and with no evidence for past barriers to genes flow. Because mt genes are tightly coadapted with N‐mt genes (Woodson and Chory 2008; Lane 2011; Burton et al. 2013), adaptive divergence in a mt gene will inevitably entail coevolution with its coadapted N‐mt genes (Hill 2014; Levin et al. 2014; Bar‐Yaacov et al. 2015). This new, coadapted mt/N‐mt complex will likely be, to some degree, incompatible with the ancestral and other coadapted mt/N‐mt complexes (Burton and Barreto 2012; Bar‐Yaacov et al. 2015). In other words, as the first step in speciation, selection can theoretically drive the coevolution of unique mt/N‐mt complexes in populations experiencing different environments that necessitate different physiological responses (Fig. 1).

This hypothesized process is best illustrated in a simple verbal model. Consider an animal species with a range that extends into different thermal environments such that there are two adaptive peaks for OXPHOS function, one associated with warmer southern environments and one associated with cooler northern environments (Fig. 1). The ancestral population has a mt gene (mt_s_) gene is best adapted for warmer environments, so all of the individuals in the northern part of the range exist away from their adaptive peak and have relatively low fitness (Fig. 1A). If a mutation occurs in the mt_s_ gene that shifts physiology to a state in which performance is enhanced in the cooler environment (mt_n_), then natural selection should lead to increasing frequency of the new, more fit mitochondrial genotype through the cooler parts of the species' range. The result would be clinal variation in mt genotype, with high proportions of mt_n_ in the north and mt_s_ in the south (Fig. 1B). With no physical barriers to gene flow, local selection would be constantly eroded by exchange of genes between subpopulations, and no subpopulation would reach a fitness peak. Such a pattern of clinal variation in mitochondrial haplotypes related to an environmental gradient is a pattern shown by wild populations (Hofman and Szymura 2007; Cheviron and Brumfield 2009; Keller and Seehausen 2012; Zakas et al. 2014; Consuegra et al. 2015).

Once there is clinal variation in COX1 genes, the interactions of mt and N‐mt genes can theoretically create barriers to gene flow that enable speciation without geographical barriers to gene flow. One can imagine that in the northern population with an overrepresentation of mt_n_, there is a mutation in a nuclear‐encoded gene (changing from the ancestral state N‐mt_0_ to N‐mt_n_) that interacts with mt_n_, making the ETC significantly better adapted to the cold climate. This novel N‐mt gene would, in effect, improve mitonuclear coadaptation in the cooler northern climate. Moreover, while this N‐mt_n_ gene is fully compatible with mt_n_, it is less compatible with (not well coadapted with) mt_s_, lowering the fitness of mt_s_/N‐mt_n_ genotypes below that of either coadapted mitonuclear genotypes (mt_s_/N‐mt_o_ or mt_n_/N‐mt_n_). This single mutation in an N‐mt gene would create a situation in which individuals with mt_n_/N‐mt_n_ genotypes have a fitness advantage in the north, individuals with mt_s_/N‐mt_0_ genes have a fitness advantage in the south, and both mt_s_/N‐mt_n_ and mt_n_/N‐mt_0_ hybrids have universal fitness disadvantages (Fig. 1C). What makes mitonuclear interactions so important to the disruption of gene flow in this hypothesized process is that hybrid dysfunction created by mitonuclear incompatibilities generate strong selection against mating between mitonuclear types and hence the potential for speciation without physical barriers to gene flow (Burton and Barreto 2012). The rapid evolution of reproductive isolation has been demonstrated in models under conditions of two locally adapted genotypes with selection against hybrids, as dictated in my verbal model (Bank et al. 2012).

Empirical studies indicate that environments imposing variable selection on components of the ETC are related to temperature, degree of hypoxia (with variation created by altitude, terrestrial versus subterranean habitats, or dissolved oxygen in water), and the need for extreme movement such as migration (reviewed in Blier et al. (2001); Das (2006); da Fonseca et al. (2008); Keller and Seehausen (2012); Hill (2015)). Once there is disruption to gene flow, selection on coadapted mitonuclear genes that enable adaptations to local abiotic environments could theoretically drive rapid change in mitochondrial genes and create the mitochondrial DNA barcode gap that is observed between even closely related species. COX genes are implicated in this process both because COX1 nucleotide sequence is recognized as the universal DNA barcode gene and because changes to COX genotype have been linked to adaptive changes in cellular respiration (Hill 2015). However, other mitochondrial genes also show evidence for adaptive divergence in response to different abiotic environments (Mishmar et al. 2006; Dowling et al. 2008; Wallace 2013; Garvin et al. 2015).

## Signatures of Adaptive Divergence in Metazoan Genomes

The relative importance of divergence in mt/N‐mt complexes driven by selection for adaptation to external environments versus by selection for maintenance mitonuclear coadaptation is currently unknown. Some evidence suggests that cases of speciation driven by adaptive divergence in COX genotype may be a small subset of all species divergences. Kwong et al. (2012) tested the hypothesis that species‐specific coadaptation of mt‐encoded COX1 gene and N‐mt‐encoded genes that code for the subunits of complex IV underlies the success of the barcode gene in delimiting species. They found little amino acid substitution between sister taxa within the portion of the COX1 nucleotide sequence used for barcoding. Based on their analysis, they concluded that there was no evidence that divergence in COX1 genotype plays any role in speciation. In detailed study of the patterns of evolution in all protein‐coding mitochondrial genes in birds, Kerr (2011) also found few amino acid substitutions between species in COX1 genes that would lead to functional change among sister taxa. These comparative studies suggest that adaptive divergence in COX1 genotype leading to speciation may be uncommon.

In a growing number of focal species studies, however, interspecific differences in the sequence of COX genes are linked to functional amino acid changes (Blier et al. 2001) and to physiological adaptations to abiotic environmental conditions (Blier and Lemieux 2001; Das 2006; Welch et al. 2014). Among sister taxa, small changes to COX genes enable adaptation to novel environments, particularly related to thermal and low‐oxygen tolerance. For instance, the Tibetan migratory locust (*Locusta migratoria*) breeds at very high elevations in Asia, and changes to its COX1 gene sequence compared with lowland locust species increased catalytic efficiency for the reduction of oxygen to water under hypoxic conditions (Zhang et al. 2013). In plateau pikas (*Ochotona curzoniae*) and Tibetan antelope (*Pantholops hodgsonii*) living at high elevations, nucleotide changes in COX1 genes also appear to code for adaptations to high‐altitude, low‐oxygen environments (Xu et al. 2005; Luo et al. 2008). Bar‐headed geese (*Anser indicus*) have a single amino acid substitution in a COX gene that improves the efficiency of oxygen use at high altitudes and enable the geese to fly over the Himalayan Mountains (Scott et al. 2011) while all other species of migratory waterfowl must fly around the mountains. Two lineages of rodents living in hypoxic subterranean habitats independently evolved a shared adaptive configuration of COX genes through different amino acid substitutions (Tomasco and Lessa 2011). Compared to Brown Bears (*Ursus arctos*), Polar Bears (*Ursus maritimus*) show evidence for increased evolution of COX1 genotype that is implicated in better tolerance of extreme cold environments (Welch et al. 2014). Adaptive changes in COX genes also underlie major adaptive radiations, including flight in bats (Shen et al. 2010) and large prey consumption in snakes (Castoe et al. 2008). These studies clearly demonstrate that changes to COX1 nucleotide sequence can underlie key physiological adaptations as well as major adaptive radiations (Hill 2015). As more genomic data come available for more sister taxa, the frequency of speciation driven by adaptive divergence versus mitonuclear coevolution not connected to environmental adaptation should become clearer.

## Conclusions and Implications

Nosil (2012) proposed two distinct processes of speciation: ecological speciation and speciation not related to environmental adaptation usually attributed to neutral divergence in allopatry. The speciation models that I propose echo this theme, but a mitonuclear coadaptation perspective provides a specific molecular model for why the two types of divergence occur. Mitonuclear coevolution to maintain coadaptation is an ubiquitous and non‐neutral process that will lead to divergence and speciation whenever populations are isolated for adequate periods of time (Gershoni et al. 2009). This process may explain most speciation events. Alternatively, populations can diverge without physical barriers to gene flow through divergent mitonuclear adaptation to the environment. This later process may best explain population divergences that seem too recent to have accommodated lineage sorting and yet accurately diagnosed with mitochondrial DNA barcodes.

The mitonuclear compatibility model of speciation proposes that when ecological speciation occurs, it will be driven by adaptation to abiotic environment. Evolutionary biologists have long mused that the patterns of biodiversity are not consistent with the idea that niche occupation and competitive exclusion dictate the number of species of animals (Hutchinson 1959). In a paper considering why there are so few species of animals, Felsenstein (1981) wrote that if sympatric speciation driven by niche partitioning were the case, then “one would expect to find nearly infinite numbers of species, a different species on every bush.” Instead, for taxa such as birds and mammals, we find a relatively few species, with sister species more likely to segregate across broad climatic and altitudinal gradients than among finer ecological niches (Hawkins et al. 2003; Currie et al. 2004). Barriers to gene flow generated by adaptation to abiotic environment will create an opportunity for niche partitioning, but by the models that I propose the rate of speciation is ultimately set by adaptation to abiotic environments via changes in mt and N‐mt coadapted complexes.

Among the most important implications of a mitonuclear compatibility species concept is that the species boundaries identified by mitochondrial DNA barcode gaps are not simply approximations of species boundaries. By this model, divergence in mitochondrial genotype is the basis for speciation, and therefore, mitochondrial genotype defines a species. To be more precise, a speciation model founded on the necessity of mitonuclear coadaptation yields a novel definition for metazoan species: *A species is a population that is reproductively isolated from other populations by incompatibilities in uniquely coadapted mt and N‐mt genes*. Species boundaries are the boundaries of coadapted mitochondrial and nuclear genes, which will be boundaries already deduced from the COX1 barcode gap. Adopting mitochondrial DNA barcode gaps as objective delineators of true species will remove subjectivity from the process of recognizing species and tangibly advance taxonomy as a science (Tautz et al. 2003; Kress et al. 2014).

## Conflict of Interest

None declared.

## References

[ece32338-bib-0001] Aliabadian, M. , M. Kaboli , V. Nijman , and M. Vences . 2009 Molecular identification of birds: performance of distance‐based DNA barcoding in three genes to delimit parapatric species. PLoS One 4:e4119. 19127298 10.1371/journal.pone.0004119 PMC2612741

[ece32338-bib-0002] Arnold, S. 2012 The power of life – cytochrome c oxidase takes center stage in metabolic control, cell signalling and survival. Mitochondrion 12:46–56. 21640202 10.1016/j.mito.2011.05.003

[ece32338-bib-0003] Baker, A. J. , E. S. Tavares , and R. F. Elbourne . 2009 Countering criticisms of single mitochondrial DNA gene barcoding in birds. Mol. Ecol. Resour. 9:257–268. 21564985 10.1111/j.1755-0998.2009.02650.x

[ece32338-bib-0004] Bank, C. , R. Bürger , and J. Hermisson . 2012 The limits to parapatric speciation: Dobzhansky‐Muller incompatibilities in a continent–island model. Genetics 191:845–863. 22542972 10.1534/genetics.111.137513 PMC3389979

[ece32338-bib-0005] Bar Yaacov, D. , K. Arbel‐Thau , Y. Zilka , O. Ovadia , A. Bouskila , and D. Mishmar . 2012 Mitochondrial DNA variation, but not nuclear DNA, sharply divides morphologically identical chameleons along an ancient geographic barrier. PLoS One 7:e31372. 22457709 10.1371/journal.pone.0031372 PMC3306244

[ece32338-bib-0006] Barreto, F. S. , and R. S. Burton . 2013 Evidence for compensatory evolution of ribosomal proteins in response to rapid divergence of mitochondrial rRNA. Mol. Biol. Evol. 30:310–314. 22993236 10.1093/molbev/mss228

[ece32338-bib-0007] Barrientos, A. , S. Müller , R. Dey , J. Wienberg , and C. T. Moraes . 2000 Cytochrome c oxidase assembly in primates is sensitive to small evolutionary variations in amino acid sequence. Mol. Biol. Evol. 17:1508–1519. 11018157 10.1093/oxfordjournals.molbev.a026250

[ece32338-bib-0008] Bar‐Yaacov, D. , A. Blumberg , and D. Mishmar . 2012 Mitochondrial‐nuclear co‐evolution and its effects on OXPHOS activity and regulation. Biochim. Biophys. Acta 1819:1107–1111. 22044624 10.1016/j.bbagrm.2011.10.008

[ece32338-bib-0009] Bar‐Yaacov, D. , Z. Hadjivasiliou , L. Levin , G. Barshad , R. Zarivach , A. Bouskila , et al. 2015 Mitochondrial involvement in vertebrate speciation? The case of mito‐nuclear genetic divergence in chameleons. Genome Biol. Evol. 7:3322–3336. 26590214 10.1093/gbe/evv226 PMC4700957

[ece32338-bib-0010] Beck, E. A. , A. C. Thompson , J. Sharbrough , E. Brud , and A. Llopart . 2015 Gene flow between *Drosophila yakuba* and *Drosophila santomea* in subunit V of cytochrome c oxidase: a potential case of cytonuclear cointrogression. Evolution 69:1973–1986. 26155926 10.1111/evo.12718 PMC5042076

[ece32338-bib-0011] Bickford, D. , D. J. Lohman , N. S. Sodhi , P. K. Ng , R. Meier , K. Winker , et al. 2007 Cryptic species as a window on diversity and conservation. Trends Ecol. Evol. 22:148–155. 17129636 10.1016/j.tree.2006.11.004

[ece32338-bib-0012] Blier, P. U. , and H. Lemieux . 2001 The impact of the thermal sensitivity of cytochrome c oxidase on the respiration rate of Arctic charr red muscle mitochondria. J. Comp. Physiol. B. 171:247–253. 11352108 10.1007/s003600000169

[ece32338-bib-0013] Blier, P. U. , F. Dufresne , and R. S. Burton . 2001 Natural selection and the evolution of mtDNA‐encoded peptides: evidence for intergenomic co‐adaptation. Trends Genet. 17:400–406. 11418221 10.1016/s0168-9525(01)02338-1

[ece32338-bib-0014] Bolnick, D. I. , M. Turelli , H. Lopez‐Fernández , P. C. Wainwright , and T. J. Near . 2008 Accelerated mitochondrial evolution and “Darwin's corollary”: asymmetric viability of reciprocal F1 hybrids in Centrarchid fishes. Genetics 178:1037–1048. 18245356 10.1534/genetics.107.081364 PMC2248366

[ece32338-bib-0015] Bonne, G. , P. Seibel , S. Possekel , C. MarSac , and B. Kadenbach . 1993 Expression of human cytochrome c oxidase subunits during fetal development. Eur. J. Biochem. 217:1099–1107. 8223633 10.1111/j.1432-1033.1993.tb18342.x

[ece32338-bib-0016] Boratyński, Z. , T. Ketola , E. Koskela , and T. Mappes . 2015 The sex specific genetic variation of energetics in bank voles: consequences of introgression? Evol. Biol. 43:37–47.

[ece32338-bib-0017] Brower, A. V. 2006 Problems with DNA barcodes for species delimitation: “ten species” of *Astraptes fulgerator* reassessed (Lepidoptera: Hesperiidae). Syst. Biodivers. 4:127–132.

[ece32338-bib-0018] Bucklin, A. , D. Steinke , and L. Blanco‐Bercial . 2011 DNA barcoding of marine metazoa. Annu. Rev. Mar. Sci. 3:471–508. 10.1146/annurev-marine-120308-080950 21329214

[ece32338-bib-0019] Burton, R. S. , and F. S. Barreto . 2012 A disproportionate role for mtDNA in Dobzhansky‐Muller incompatibilities? Mol. Ecol. 21:4942–4957. 22994153 10.1111/mec.12006

[ece32338-bib-0020] Burton, R. S. , R. J. Pereira , and F. S. Barreto . 2013 Cytonuclear genomic interactions and hybrid breakdown. Annu. Rev. Ecol. Evol. Syst. 44:281–302.

[ece32338-bib-0021] Butlin, R. , A. Debelle , C. Kerth , R. Snook , L. Beukeboom , R. Castillo Cajas , et al. 2012 What do we need to know about speciation? Trends Ecol. Evol. 27:27–39. 21978464 10.1016/j.tree.2011.09.002

[ece32338-bib-0022] Calvo, S. E. , and V. K. Mootha . 2010 The mitochondrial proteome and human disease. Annu. Rev. Genomics Hum. Genet. 11:25–44. 20690818 10.1146/annurev-genom-082509-141720 PMC4397899

[ece32338-bib-0023] Castoe, T. A. , Z. J. Jiang , W. Gu , Z. O. Wang , and D. D. Pollock . 2008 Adaptive evolution and functional redesign of core metabolic proteins in snakes. PLoS One 3:e2201. 18493604 10.1371/journal.pone.0002201 PMC2376058

[ece32338-bib-0024] Charlat, S. , A. Duplouy , E. A. Hornett , E. A. Dyson , N. Davies , G. K. Roderick , et al. 2009 The joint evolutionary histories of *Wolbachia* and mitochondria in *Hypolimnas bolina* . BMC Evol. Biol. 9:1. 19317891 10.1186/1471-2148-9-64 PMC2669805

[ece32338-bib-0025] Cheviron, Z. A. , and R. T. Brumfield . 2009 Migration‐selection balance and local adaptation of mitochondrial haplotypes in rufous‐collared sparrows (*Zonotrichia capensis*) along an elevational gradient. Evolution 63:1593–1605. 19187247 10.1111/j.1558-5646.2009.00644.x

[ece32338-bib-0026] Chou, J. Y. , and J. Y. Leu . 2010 Speciation through cytonuclear incompatibility: insights from yeast and implications for higher eukaryotes. BioEssays 32:401–411. 20414898 10.1002/bies.200900162

[ece32338-bib-0027] Chou, J.‐Y. , and J.‐Y. Leu . 2015 The Red Queen in mitochondria: cyto‐nuclear co‐evolution, hybrid breakdown and human disease. Front. Genet. 6:187. 26042149 10.3389/fgene.2015.00187 PMC4437034

[ece32338-bib-0028] Consuegra, S. , E. John , E. Verspoor , and C. G. de Leaniz . 2015 Patterns of natural selection acting on the mitochondrial genome of a locally adapted fish species. Genet. Sel. Evol. 47:1–10. 26138253 10.1186/s12711-015-0138-0 PMC4490732

[ece32338-bib-0029] Coyne, J. A. , and H. A. Orr . 2004 Speciation. Sinauer Associates Inc, New York.

[ece32338-bib-0030] Crow, J. F. , and M. Kimura . 2009 An introduction to population genetics theory. Blackburn Press, Caldwell, NJ.

[ece32338-bib-0031] Currie, D. J. , G. G. Mittelbach , H. V. Cornell , R. Field , J. Guégan , B. A. Hawkins , et al. 2004 Predictions and tests of climate‐based hypotheses of broad‐scale variation in taxonomic richness. Ecol. Lett. 7:1121–1134.

[ece32338-bib-0032] Das, J. 2006 The role of mitochondrial respiration in physiological and evolutionary adaptation. BioEssays 28:890–901. 16937356 10.1002/bies.20463

[ece32338-bib-0033] Dasmahapatra, K. , and J. Mallet . 2006 DNA barcodes: recent successes and future prospects. Heredity 97:254–255. 16788705 10.1038/sj.hdy.6800858

[ece32338-bib-0034] Deagle, B. E. , S. N. Jarman , E. Coissac , F. Pompanon , and P. Taberlet . 2014 DNA metabarcoding and the cytochrome c oxidase subunit I marker: not a perfect match. Biol. Lett. 10:20140562. 25209199 10.1098/rsbl.2014.0562 PMC4190964

[ece32338-bib-0035] Dobzhansky, T. 1936 Studies on hybrid sterility. II. Localization of sterility factors in *Drosophila pseudoobscura* hybrids. Genetics 21:113. 17246786 10.1093/genetics/21.2.113 PMC1208664

[ece32338-bib-0036] Dowling, D. K. , U. Friberg , and J. Lindell . 2008 Evolutionary implications of non‐neutral mitochondrial genetic variation. Trends Ecol. Evol. 23:546–554. 18722688 10.1016/j.tree.2008.05.011

[ece32338-bib-0037] Dubois, A. 2007 Phylogeny, taxonomy and nomenclature: the problem of taxonomic categories and of nomenclatural ranks. Zootaxa 1519:27–68.

[ece32338-bib-0038] Eberhardt, U. 2012 Methods for DNA barcoding of fungi Pp. 183–206 *in* Kress W. J. and Erickson D. L., eds. DNA barcodes: methods and protocols: methods in molecular biology. Humana Press, New York. 10.1007/978-1-61779-591-6_9 22684957

[ece32338-bib-0039] Elias, M. , R. I. Hill , K. R. Willmott , K. K. Dasmahapatra , A. V. Brower , J. Mallet , et al. 2007 Limited performance of DNA barcoding in a diverse community of tropical butterflies. Proc. Biol. Sci. 274:2881–2889. 17785265 10.1098/rspb.2007.1035 PMC3227132

[ece32338-bib-0040] Ellison, C. K. , and R. S. Burton . 2006 Disruption of mitochondrial function in interpopulation hybrids of *Tigriopus californicus* . Evolution 60:1382–1391. 16929655

[ece32338-bib-0041] Ellison, C. K. , and R. S. Burton . 2008 Interpopulation hybrid breakdown maps to the mitochondrial genome. Evolution 62:631–638. 18081717 10.1111/j.1558-5646.2007.00305.x

[ece32338-bib-0042] Ellison, C. , O. Niehuis , and J. Gadau . 2008 Hybrid breakdown and mitochondrial dysfunction in hybrids of *Nasonia* parasitoid wasps. J. Evol. Biol. 21:1844–1851. 18811665 10.1111/j.1420-9101.2008.01608.x

[ece32338-bib-0043] Felsenstein, J. . 1981 Skepticism towards Santa Rosalia, or why are there so few kinds of animals? Evolution 35:124–138. 10.1111/j.1558-5646.1981.tb04864.x 28563447

[ece32338-bib-0044] da Fonseca, R. R. , W. E. Johnson , S. J. O'Brien , M. J. Ramos , and A. Antunes . 2008 The adaptive evolution of the mammalian mitochondrial genome. BMC Genom. 9:119. 10.1186/1471-2164-9-119 PMC2375446 18318906

[ece32338-bib-0045] Fujisawa, T. , and T. G. Barraclough . 2013 Delimiting species using single‐locus data and the Generalized Mixed Yule Coalescent (GMYC) approach: a revised method and evaluation on simulated datasets. Syst. Biol. 62:707–724. 23681854 10.1093/sysbio/syt033 PMC3739884

[ece32338-bib-0046] Garvin, M. R. , J. P. Bielawski , L. A. Sazanov , and A. J. Gharrett . 2015 Review and meta‐analysis of natural selection in mitochondrial complex I in metazoans. J. Zoolog. Syst. Evol. Res. 53:1–17.

[ece32338-bib-0047] Gershoni, M. , A. R. Templeton , and D. Mishmar . 2009 Mitochondrial bioenergetics as a major motive force of speciation. BioEssays 31:642–650. 19408245 10.1002/bies.200800139

[ece32338-bib-0048] Harrison, J. S. , and R. S. Burton . 2006 Tracing hybrid incompatibilities to single amino acid substitutions. Mol. Biol. Evol. 23:559–564. 16280539 10.1093/molbev/msj058

[ece32338-bib-0049] Havird, J. C. , N. S. Whitehill , C. D. Snow , and D. B. Sloan . 2015 Conservative and compensatory evolution in oxidative phosphorylation complexes of angiosperms with highly divergent rates of mitochondrial genome evolution. Evolution 69:3069–3081. 26514987 10.1111/evo.12808 PMC4715514

[ece32338-bib-0050] Hawkins, B. A. , E. E. Porter , and J. A. Felizola Diniz‐Filho . 2003 Productivity and history as predictors of the latitudinal diversity gradient of terrestrial birds. Ecology 84:1608–1623.

[ece32338-bib-0051] Hebert, P. D. , A. Cywinska , and S. L. Ball . 2003a Biological identifications through DNA barcodes. Proc. Biol. Sci. 270:313–321. 12614582 10.1098/rspb.2002.2218 PMC1691236

[ece32338-bib-0052] Hebert, P. D. , S. Ratnasingham , and J. R. de Waard . 2003b Barcoding animal life: cytochrome c oxidase subunit 1 divergences among closely related species. Proc. Biol. Sci. 270:S96–S99. 12952648 10.1098/rsbl.2003.0025 PMC1698023

[ece32338-bib-0053] Hebert, P. D. , E. H. Penton , J. M. Burns , D. H. Janzen , and W. Hallwachs . 2004 Ten species in one: DNA barcoding reveals cryptic species in the neotropical skipper butterfly *Astraptes fulgerator* . Proc. Natl Acad. Sci. USA 101:14812–14817. 15465915 10.1073/pnas.0406166101 PMC522015

[ece32338-bib-0054] Hickerson, M. J. , C. P. Meyer , and C. Moritz . 2006 DNA barcoding will often fail to discover new animal species over broad parameter space. Syst. Biol. 55:729–739. 17060195 10.1080/10635150600969898

[ece32338-bib-0055] Hill, G. E. 2014 Sex linkage of nuclear‐encoded mitochondrial genes. Heredity 112:469–470. 24346499 10.1038/hdy.2013.125 PMC3998780

[ece32338-bib-0056] Hill, G. E. 2015 Mitonuclear ecology. Mol. Biol. Evol. 32:1917–1927. 25931514 10.1093/molbev/msv104 PMC4833085

[ece32338-bib-0057] Hofman, S. , and J. M. Szymura . 2007 Limited mitochondrial DNA introgression in a Bombina hybrid zone. Biol. J. Linn. Soc. 91:295–306.

[ece32338-bib-0058] Hudson, R. R. , and J. A. Coyne . 2002 Mathematical consequences of the genealogical species concept. Evolution 56:1557–1565. 12353748 10.1111/j.0014-3820.2002.tb01467.x

[ece32338-bib-0059] Hudson, R. R. , and M. Turelli . 2003 Stochasticity overrules the “three‐times rule”: genetic drift, genetic draft, and coalescence times for nuclear loci versus mitochondrial DNA. Evolution 57:182–190. 12643581 10.1111/j.0014-3820.2003.tb00229.x

[ece32338-bib-0060] Hurst, G. D. , and F. M. Jiggins . 2005 Problems with mitochondrial DNA as a marker in population, phylogeographic and phylogenetic studies: the effects of inherited symbionts. Proc. Biol. Sci. 272:1525–1534. 16048766 10.1098/rspb.2005.3056 PMC1559843

[ece32338-bib-0061] Hutchinson, G. E. . 1959 Homage to Santa Rosalia or why are there so many kinds of animals? Am. Nat. 93:145–159.

[ece32338-bib-0062] Hüttemann, M. , B. Kadenbach , and L. I. Grossman . 2001 Mammalian subunit IV isoforms of cytochrome c oxidase. Gene 267:111–123. 11311561 10.1016/s0378-1119(01)00385-7

[ece32338-bib-0063] Kadenbach, B. , M. Hüttemann , S. Arnold , I. Lee , and E. Bender . 2000 Mitochondrial energy metabolism is regulated via nuclear‐coded subunits of cytochrome c oxidase. Free Radic. Biol. Med. 29:211–221. 11035249 10.1016/s0891-5849(00)00305-1

[ece32338-bib-0064] Keller, I. , and O. Seehausen . 2012 Thermal adaptation and ecological speciation. Mol. Ecol. 21:782–799. 22182048 10.1111/j.1365-294X.2011.05397.x

[ece32338-bib-0065] Kenyon, L. , and C. T. Moraes . 1997 Expanding the functional human mitochondrial DNA database by the establishment of primate xenomitochondrial cybrids. Proc. Natl Acad. Sci. USA 94:9131–9135. 9256447 10.1073/pnas.94.17.9131 PMC23071

[ece32338-bib-0066] Kerr, K. C. 2011 Searching for evidence of selection in avian DNA barcodes. Mol. Ecol. Resour. 11:1045–1055. 21777399 10.1111/j.1755-0998.2011.03049.x

[ece32338-bib-0067] Kerr, K. C. , M. Y. Stoeckle , C. J. Dove , L. A. Weigt , C. M. Francis , and P. D. Hebert . 2007 Comprehensive DNA barcode coverage of North American birds. Mol. Ecol. Notes 7:535–543. 18784793 10.1111/j.1471-8286.2007.01670.x PMC2259444

[ece32338-bib-0068] Kerr, K. C. , S. M. Birks , M. V. Kalyakin , Y. A. Red'kin , E. A. Koblik , and P. D. Hebert . 2009a Filling the gap‐COI barcode resolution in eastern Palearctic birds. Front. Zool. 6:29–42. 20003213 10.1186/1742-9994-6-29 PMC2796652

[ece32338-bib-0069] Kerr, K. C. , D. A. Lijtmaer , A. S. Barreira , P. D. Hebert , and P. L. Tubaro . 2009b Probing evolutionary patterns in Neotropical birds through DNA barcodes. PLoS One 4:e4379. 19194495 10.1371/journal.pone.0004379 PMC2632745

[ece32338-bib-0070] Klopfstein, S. , C. Kropf , and H. Baur . 2016 Wolbachia endosymbionts distort DNA barcoding in the parasitoid wasp genus *Diplazon* (Hymenoptera: Ichneumonidae). Zool. J. Linn. Soc. 177:541–557.

[ece32338-bib-0071] Kress, W. J. , K. J. Wurdack , E. A. Zimmer , L. A. Weigt , and D. H. Janzen . 2005 Use of DNA barcodes to identify flowering plants. Proc. Natl Acad. Sci. USA 102:8369–8374. 15928076 10.1073/pnas.0503123102 PMC1142120

[ece32338-bib-0072] Kress, W. J. , C. García‐Robledo , M. Uriarte , and D. L. Erickson . 2014 DNA barcodes for ecology, evolution, and conservation. Trends Ecol. Evol. 30:25–35. 25468359 10.1016/j.tree.2014.10.008

[ece32338-bib-0073] Kwong, S. , A. Srivathsan , G. Vaidya , and R. Meier . 2012 Is the COI barcoding gene involved in speciation through intergenomic conflict? Mol. Phylogenet. Evol. 62:1009–1012. 22182989 10.1016/j.ympev.2011.11.034

[ece32338-bib-0074] Lane, N. 2009 On the origin of bar codes. Nature 462:272–274. 19924185 10.1038/462272a

[ece32338-bib-0075] Lane, N. 2011 Mitonuclear match: optimizing fitness and fertility over generations drives ageing within generations. BioEssays 33:860–869. 21922504 10.1002/bies.201100051

[ece32338-bib-0076] Lane, N. 2015 The vital question: energy, evolution, and the origins of complex life. W. W. Norton & Company, New York.

[ece32338-bib-0077] Levin, L. , A. Blumberg , G. Barshad , and D. Mishmar . 2014 Mito‐nuclear co‐evolution: the positive and negative sides of functional ancient mutations. Front. Genet. 5:448. 25566330 10.3389/fgene.2014.00448 PMC4274989

[ece32338-bib-0078] Lohse, K. . 2009 Can mtDNA barcodes be used to delimit species? A response to Pons et al. (2006). Syst. Biol. 58:439–442. 20525596 10.1093/sysbio/syp039

[ece32338-bib-0079] Luo, Y. , W. Gao , Y. Gao , S. Tang , Q. Huang , X. Tan , et al. 2008 Mitochondrial genome analysis of *Ochotona curzoniae* and implication of cytochrome c oxidase in hypoxic adaptation. Mitochondrion 8:352–357. 18722554 10.1016/j.mito.2008.07.005

[ece32338-bib-0080] Lynch, M. 2010 Evolution of the mutation rate. Trends Genet. 26:345–352. 20594608 10.1016/j.tig.2010.05.003 PMC2910838

[ece32338-bib-0081] Lynch, M. , and J. L. Blanchard . 1998 Deleterious mutation accumulation in organelle genomes. Genetica 102:29–39. 9720269

[ece32338-bib-0082] Mayr, E. 1942 Systematics and the origin of species. Columbia Univ. Press, New York.

[ece32338-bib-0083] McKenzie, M. , M. Chiotis , C. A. Pinkert , and I. A. Trounce . 2003 Functional respiratory chain analyses in murid xenomitochondrial cybrids expose coevolutionary constraints of cytochrome b and nuclear subunits of complex III. Mol. Biol. Evol. 20:1117–1124. 12777531 10.1093/molbev/msg132

[ece32338-bib-0084] Meier, R. , K. Shiyang , G. Vaidya , and P. K. Ng . 2006 DNA barcoding and taxonomy in Diptera: a tale of high intraspecific variability and low identification success. Syst. Biol. 55:715–728. 17060194 10.1080/10635150600969864

[ece32338-bib-0085] Meiklejohn, C. D. , M. A. Holmbeck , M. A. Siddiq , D. N. Abt , D. M. Rand , and K. L. Montooth . 2013 An incompatibility between a mitochondrial tRNA and its nuclear‐encoded tRNA synthetase compromises development and fitness in *Drosophila* . PLoS Genet. 9:e1003238. 23382693 10.1371/journal.pgen.1003238 PMC3561102

[ece32338-bib-0086] Mishmar, D. , E. Ruiz‐Pesini , M. Mondragon‐Palomino , V. Procaccio , B. Gaut , and D. C. Wallace . 2006 Adaptive selection of mitochondrial complex I subunits during primate radiation. Gene 378:11–18. 16828987 10.1016/j.gene.2006.03.015

[ece32338-bib-0087] Moritz, C. , and C. Cicero . 2004 DNA barcoding: promise and pitfalls. PLoS Biol. 2:e354. 15486587 10.1371/journal.pbio.0020354 PMC519004

[ece32338-bib-0088] Morrison, W. , J. Lohr , P. Duchen , R. Wilches , D. Trujillo , M. Mair , et al. 2009 The impact of taxonomic change on conservation: does it kill, can it save, or is it just irrelevant? Biol. Conserv. 142:3201–3206.

[ece32338-bib-0089] Muller, H. J. 1942 Isolating mechanisms, evolution and temperature Pp. 71–125 *in* Dobzhansky T., ed. Biological symposia: a series of volumes devoted to current symposia in the field of biology (Vol. 6). Jaques Cattell Press, Lancaster, PA.

[ece32338-bib-0090] Mutanen, M. , S. M. Kivelä , R. A. Vos , C. Doorenweerd , S. Ratnasingham , A. Hausmann , et al. in press. Species level Para‐and Polyphyly in DNA Barcode Gene Trees: strong operational bias in European Lepidoptera. Syst. Biol. doi:10.1093/sysbio/syw044. 10.1093/sysbio/syw044 PMC5066064 27288478

[ece32338-bib-0091] Nei, M. 1987 Molecular evolutionary genetics. Columbia Univ. Press, Cary, NC, USA.

[ece32338-bib-0092] Neiman, M. , and D. R. Taylor . 2009 The causes of mutation accumulation in mitochondrial genomes. Proc. Biol. Sci. 276:1201–1209. 19203921 10.1098/rspb.2008.1758 PMC2660971

[ece32338-bib-0093] Nosil, P. 2012 Ecological speciation. Oxford Univ. Press, Cary, NC, USA.

[ece32338-bib-0094] Pacelli, C. , D. Latorre , T. Cocco , F. Capuano , C. Kukat , P. Seibel , et al. 2011 Tight control of mitochondrial membrane potential by cytochrome c oxidase. Mitochondrion 11:334–341. 21147274 10.1016/j.mito.2010.12.004

[ece32338-bib-0095] Pesole, G. , C. Gissi , A. De Chirico , and C. Saccone . 1999 Nucleotide substitution rate of mammalian mitochondrial genomes. J. Mol. Evol. 48:427–434. 10079281 10.1007/pl00006487

[ece32338-bib-0096] Piccoli, C. , R. Scrima , D. Boffoli , and N. Capitanio . 2006 Control by cytochrome c oxidase of the cellular oxidative phosphorylation system depends on the mitochondrial energy state. Biochem. J. 396:573–583. 16533168 10.1042/BJ20060077 PMC1482809

[ece32338-bib-0097] Pierron, D. , D. E. Wildman , M. Hüttemann , G. C. Markondapatnaikuni , S. Aras , and L. I. Grossman . 2012 Cytochrome c oxidase: evolution of control via nuclear subunit addition. Biochim. Biophys. Acta 1817:590–597. 21802404 10.1016/j.bbabio.2011.07.007 PMC3923406

[ece32338-bib-0098] Price, T. 2007 Speciation in birds. Roberts and Company Publishers, London.

[ece32338-bib-0099] vRamzan, R. , P. Weber , B. Kadenbach , and S. Vogt . 2012 Individual biochemical behaviour versus biological robustness: spotlight on the regulation of cytochrome c oxidase Pp. 265–281 *in* Mitochondrial oxidative phosphorylation. Springer, New York, NY. 10.1007/978-1-4614-3573-0_11 22729862

[ece32338-bib-0100] Rand, D. M. , R. A. Haney , and A. J. Fry . 2004 Cytonuclear coevolution: the genomics of cooperation. Trends Ecol. Evol. 19:645–653. 16701327 10.1016/j.tree.2004.10.003

[ece32338-bib-0101] Rawson, P. D. , and R. S. Burton . 2002 Functional coadaptation between cytochrome c and cytochrome c oxidase within allopatric populations of a marine copepod. Proc. Natl Acad. Sci. USA 99:12955–12958. 12271133 10.1073/pnas.202335899 PMC130567

[ece32338-bib-0102] Remsen, J. Jr , K. Winker , and S. Haig . 2010 Subspecies as a meaningful taxonomic rank in avian classification. Ornithol. Monogr. 67:62–78.

[ece32338-bib-0103] Rubinoff, D. , S. Cameron , and K. Will . 2006 A genomic perspective on the shortcomings of mitochondrial DNA for “barcoding” identification. J. Hered. 97:581–594. 17135463 10.1093/jhered/esl036

[ece32338-bib-0104] Sackton, T. B. , R. A. Haney , and D. M. Rand . 2003 Cytonuclear coadaptation in *Drosophila*: disruption of cytochrome c oxidase activity in backcross genotypes. Evolution 57:2315–2325. 14628919 10.1111/j.0014-3820.2003.tb00243.x

[ece32338-bib-0105] Scheffers, B. R. , L. N. Joppa , S. L. Pimm , and W. F. Laurance . 2012 What we know and don't know about Earth's missing biodiversity. Trends Ecol. Evol. 27:501–510. 22784409 10.1016/j.tree.2012.05.008

[ece32338-bib-0106] Scott, G. R. , P. M. Schulte , S. Egginton , A. L. Scott , J. G. Richards , and W. K. Milsom . 2011 Molecular evolution of cytochrome c oxidase underlies high‐altitude adaptation in the bar‐headed goose. Mol. Biol. Evol. 28:351–363. 20685719 10.1093/molbev/msq205

[ece32338-bib-0107] Seberg, O. , C. J. Humphries , S. Knapp , D. W. Stevenson , G. Petersen , N. Scharff , et al. 2003 Shortcuts in systematics? A commentary on DNA‐based taxonomy. Trends Ecol. Evol. 18:63–65.

[ece32338-bib-0108] Shen, Y.‐Y. , L. Liang , Z.‐H. Zhu , W.‐P. Zhou , D. M. Irwin , and Y.‐P. Zhang . 2010 Adaptive evolution of energy metabolism genes and the origin of flight in bats. Proc. Natl Acad. Sci. USA 107:8666–8671. 20421465 10.1073/pnas.0912613107 PMC2889356

[ece32338-bib-0109] van der Sluis, E. O. , H. Bauerschmitt , T. Becker , T. Mielke , J. Frauenfeld , O. Berninghausen , et al. 2015 Parallel structural evolution of mitochondrial ribosomes and OXPHOS complexes. Genome Biol. Evol. 7:1235–1251. 25861818 10.1093/gbe/evv061 PMC4453056

[ece32338-bib-0110] Smith, M. A. , C. Bertrand , K. Crosby , E. S. Eveleigh , J. Fernandez‐Triana , B. L. Fisher , et al. 2012 *Wolbachia* and DNA barcoding insects: patterns, potential, and problems. PLoS One 7:e36514. 22567162 10.1371/journal.pone.0036514 PMC3342236

[ece32338-bib-0111] Smith, B. T. , J. E. McCormack , A. M. Cuervo , M. J. Hickerson , A. Aleixo , C. D. Cadena , et al. 2014 The drivers of tropical speciation. Nature 515:406–409. 25209666 10.1038/nature13687

[ece32338-bib-0112] Stoeckle, M. Y. , and D. S. Thaler . 2014 DNA barcoding works in practice but not in (neutral) theory. PLoS One 9:e100755. 24988408 10.1371/journal.pone.0100755 PMC4079456

[ece32338-bib-0113] Tautz, D. , P. Arctander , A. Minelli , R. H. Thomas , and A. P. Vogler . 2003 A plea for DNA taxonomy. Trends Ecol. Evol. 18:70–74.

[ece32338-bib-0114] Tavares, E. S. , and A. J. Baker . 2008 Single mitochondrial gene barcodes reliably identify sister‐species in diverse clades of birds. BMC Evol. Biol. 8:14. 18328107 10.1186/1471-2148-8-81 PMC2279116

[ece32338-bib-0115] Tavares, E. S. , P. Gonçalves , C. Y. Miyaki , and A. J. Baker . 2011 DNA barcode detects high genetic structure within Neotropical bird species. PLoS One 6:e28543. 22163311 10.1371/journal.pone.0028543 PMC3233584

[ece32338-bib-0116] Toews, D. P. , and A. Brelsford . 2012 The biogeography of mitochondrial and nuclear discordance in animals. Mol. Ecol. 21:3907–3930. 22738314 10.1111/j.1365-294X.2012.05664.x

[ece32338-bib-0117] Tomasco, I. H. , and E. P. Lessa . 2011 The evolution of mitochondrial genomes in subterranean caviomorph rodents: adaptation against a background of purifying selection. Mol. Phylogenet. Evol. 61:64–70. 21723951 10.1016/j.ympev.2011.06.014

[ece32338-bib-0118] Villani, G. , and G. Attardi . 1997 In vivo control of respiration by cytochrome c oxidase in wild‐type and mitochondrial DNA mutation‐carrying human cells. Proc. Natl Acad. Sci. USA 94:1166–1171. 9037024 10.1073/pnas.94.4.1166 PMC19762

[ece32338-bib-0119] Wallace, D. C. 2010 Mitochondrial DNA mutations in disease and aging. Environ. Mol. Mutagen. 51:440–450. 20544884 10.1002/em.20586

[ece32338-bib-0120] Wallace, D. C. 2013 Bioenergetics in human evolution and disease: implications for the origins of biological complexity and the missing genetic variation of common diseases. Philos. Trans. R. Soc. Lond. B Biol. Sci. 368:20120267. 23754818 10.1098/rstb.2012.0267 PMC3685467

[ece32338-bib-0121] Weir, J. T. , and D. Schluter . 2007 The latitudinal gradient in recent speciation and extinction rates of birds and mammals. Science 315:1574–1576. 17363673 10.1126/science.1135590

[ece32338-bib-0122] Welch, A. J. , O. C. Bedoya‐Reina , L. Carretero‐Paulet , W. Miller , K. D. Rode , and C. Lindqvist . 2014 Polar bears exhibit genome‐wide signatures of bioenergetic adaptation to life in the arctic environment. Genome Biol. Evol. 6:433–450. 24504087 10.1093/gbe/evu025 PMC3942037

[ece32338-bib-0123] Whitworth, T. , R. Dawson , H. Magalon , and E. Baudry . 2007 DNA barcoding cannot reliably identify species of the blowfly genus *Protocalliphora* (Diptera: Calliphoridae). Proc. Biol. Sci. 274:1731–1739. 17472911 10.1098/rspb.2007.0062 PMC2493573

[ece32338-bib-0124] Will, K. W. , B. D. Mishler , and Q. D. Wheeler . 2005 The perils of DNA barcoding and the need for integrative taxonomy. Syst. Biol. 54:844–851. 16243769 10.1080/10635150500354878

[ece32338-bib-0125] Woodson, J. D. , and J. Chory . 2008 Coordination of gene expression between organellar and nuclear genomes. Nat. Rev. Genet. 9:383–395. 18368053 10.1038/nrg2348 PMC4854206

[ece32338-bib-0126] Xu, S. Q. , Y. Z. Yang , J. Zhou , G. E. Jing , Y. T. Chen , J. Wang , et al. 2005 A mitochondrial genome sequence of the Tibetan antelope (*Pantholops hodgsonii*). Genomics Proteomics Bioinformatics 3:5–17. 16144518 10.1016/S1672-0229(05)03003-2 PMC5172476

[ece32338-bib-0127] Yu, D. W. , Y. Ji , B. C. Emerson , X. Wang , C. Ye , C. Yang , et al. 2012 Biodiversity soup: metabarcoding of arthropods for rapid biodiversity assessment and biomonitoring. Methods Ecol. Evol. 3:613–623.

[ece32338-bib-0128] Zakas, C. , K. Jones , and J. P. Wares . 2014 Homogeneous nuclear background for mitochondrial cline in Northern range of *Notochthamalus scabrosus* . G3 4:225–230. 24347623 10.1534/g3.113.008383 PMC3931557

[ece32338-bib-0129] Zhang, Z.‐Y. , B. Chen , D.‐J. Zhao , and L. Kang . 2013 Functional modulation of mitochondrial cytochrome c oxidase underlies adaptation to high‐altitude hypoxia in a Tibetan migratory locust. Proc. Biol. Sci. 280:20122758. 23390104 10.1098/rspb.2012.2758 PMC3574369

